# Time to revisit the use of G-CSF after allogeneic haematopoietic cell transplantation in COVID-19 era?

**DOI:** 10.1038/s41416-020-01195-8

**Published:** 2021-01-04

**Authors:** Alexandre E. Malek

**Affiliations:** Department of Infectious Diseases, Infection Control and Employee Health, The University of Texas MD Anderson Cancer Center, 1515 Holcombe Blvd., Houston, TX 77030 USA

**Keywords:** Cancer stem cells, Pathogenesis

## Abstract

The use of granulocyte colony-stimulating factor (G-CSF) in patients with haematological malignancies is associated with less febrile neutropenia episodes. But in the presence of COVID-19 infection, the administration of G-CSF is challenging as it may trigger a robust inflammatory reaction resulting in cytokine storm, respiratory failure and severe outcomes.

## Main

Although the administration of granulocyte colony-stimulating factor (G-CSF) after haematopoietic cell transplantation (HCT) has been shown to accelerate the recovery of neutrophil count and reduce febrile neutropenia episodes, its use has remained controversial with conflicting benefits on the clinical outcomes and the length of hospitalisation. Additionally, the use of G-CSF after allogeneic HCT is not well established, with heightened risk of graft-versus-host disease.^1–3^ G-CSF is a growth factor that can stimulate the expression of interleukin-1 (IL-1), interleukin-6 (IL-6), tumour necrosis factor (TNF)-α mediators and myeloid cell recovery, leading to a potential exuberant inflammatory response during the immune reconstitution and engraftment after transplantation.

This raises concerns for worrisome prognosis among HCT recipients who are contracting severe acute respiratory syndrome coronavirus 2 following HCT primarily during the pre-engraftment period. Given the rapid spread of novel coronavirus 2019 pandemic (COVID-19), confirmed COVID-19 cases have reached more than 31,000,000 worldwide with 960,000 deaths.^4^ Therefore, more HCT patients are at greater risk of infections and the immunopathogenesis of COVID-19 implicates a virus-driven organ damage followed by a robust inflammatory reaction involving the overexpression of several proinflammatory mediators such as IL-6, TNF and host G-CSF resulting in a cytokine storm that contributes to further vital organ compromise, multisystem dysfunction syndrome and death.^5^

All in all, concurrent COVID-19 infection in HCT recipients receiving G-CSF therapy can potentially lead to deleterious results and poor clinical outcomes. Further studies and clinical data are timely needed to evaluate the safety and benefits of using G-CSF following HCT during COVID-19 pandemic, as controlling the hyperinflammation response secondary to COVID-19 infection is a cornerstone in the management.

## Data Availability

Not applicable.
